# Identification of Differentially Expressed MicroRNAs in Osteosarcoma

**DOI:** 10.1155/2011/732690

**Published:** 2011-07-18

**Authors:** Rishi R. Lulla, Fabricio F. Costa, Jared M. Bischof, Pauline M. Chou, Maria de F. Bonaldo, Elio F. Vanin, Marcelo B. Soares

**Affiliations:** ^1^Division of Hematology, Oncology and Stem Cell Transplantation, Children's Memorial Hospital, 2300 Children's Plaza, Chicago, IL 60614, USA; ^2^Department of Pediatrics, Feinberg School of Medicine Northwestern University, Chicago, IL 60611, USA; ^3^Cancer Biology and Epigenomics Program, Children's Memorial Research Center, Chicago, IL 60614, USA; ^4^Division of Anatomic Pathology, Children's Memorial Hospital, Chicago, IL 60614, USA

## Abstract

A limited number of reports have investigated the role of microRNAs in osteosarcoma. In this study, we performed miRNA expression profiling of osteosarcoma cell lines, tumor samples, and normal human osteoblasts. Twenty-two differentially expressed microRNAs were identified using high throughput real-time PCR analysis, and 4 (miR-135b, miR-150, miR-542-5p, and miR-652) were confirmed and validated in a different group of tumors. Both miR-135b and miR-150 have been previously shown to be important in cancer. We hypothesize that dysregulation of differentially expressed microRNAs may contribute to tumorigenesis. They might also represent molecular biomarkers or targets for drug development in osteosarcoma.

## 1. Introduction

Osteosarcoma is the most common malignant bone tumor in childhood and adolescence. Although many tumors initially respond to chemotherapy, patients with metastatic disease and/or relapsed disease continue to have extremely poor survival outcomes [1]. A better understanding of osteosarcoma biology is needed to further optimize treatment strategies, biomarker identification, and the development of new chemotherapeutic agents. Previous studies have demonstrated diverse genetic alterations in osteosarcoma cells, including structural abnormalities, gains and/or losses of chromosomes, as well as mutations in tumor suppressor genes [2]. Epigenetic modifications such as genomic DNA methylation and alterations of chromatin-associated proteins have also been implicated in osteosarcoma carcinogenesis [3, 4]. However, the role of noncoding RNAs, especially microRNAs, in the initiation and progression of osteosarcoma is yet to be elucidated. 

MicroRNAs (miRNAs) are small noncoding RNAs that can regulate gene expression by blocking mRNA translation and/or affecting mRNA stability within cells [5]. In the last few years, there has been growing evidence that miRNAs are key regulators in the cells; a single miRNA can affect the expression of hundreds of protein coding target genes [6]. Dysregulated miRNA expression has been demonstrated in many human disease states, including cancer. Up- and/or downregulation of miRNA expression in cancer suggest that miRNAs can function as classical tumor suppressor genes or oncogenes [7, 8]. Further, miRNAs have been implicated in the development of tumor metastasis [9]. Though previous publications have explored the role of miRNAs in many adult and pediatric cancers, there is limited understanding of their role in osteosarcoma. 

A study of osteosarcoma cell lines and primary tumor samples revealed an interaction between miR-34 and p53; tumor samples showed a decreased expression of miR-34 and inhibited p53-mediated cell cycle arrest and apoptosis [10]. In another study using osteosarcoma cells in a mouse xenograft model, increased miR-140 expression was associated with chemoresistance [11]. The same group recently reported that miR-215 conferred chemoresistance to methotrexate in osteosarcoma cells *in vitro* by suppression of a cell cycle-regulated nuclear and centrosome protein [12]. In a study using 8 paired tumor and normal tissue samples as well as the osteosarcoma cell line MG-63, Ziyan et al. demonstrated upregulation of miR-21 (a well-known oncomiR in other tumor types) in osteosarcoma [13]. Knockdown of miR-21 in the cell line resulted in decreased cell invasion and migration [13]. Another group identified a proapoptotic function of miR-143 through downregulation of Bcl-2 expression in osteosarcoma cell lines and primary tumor samples [14]. 

The goal of this study was to identify differentially expressed miRNAs in osteosarcoma cell lines and tumor samples. We screened 762 mature miRNAs in 2 osteosarcoma cell lines and 4 formalin-fixed paraffin-embedded (FFPE) osteosarcoma samples. We have confirmed the expression of selected miRNAs using real-time quantitative PCR (RT Q-PCR) in the initial samples and validated the findings in 3 additional FFPE tumors. These findings provided some insights into the role of miRNAs in osteosarcoma and might be of importance for the identification of new biomarkers and future drug design.

## 2. Materials and Methods

### 2.1. Patient Samples

After approval by the Institutional Review Board (IRB) at Children's Memorial Hospital, a total of 10 primary nondecalcified FFPE blocks were obtained from pediatric patients with osteosarcoma treated at our institution between 1997 and 2010. In all cases, the final pathologic diagnosis was conventional osteoblastic osteosarcoma and the patients had no previous treatment with chemotherapy. From each tumor block, a representative H & E slide was prepared and 5–20-*μ*m thick sections were cut and provided for further analysis. 

### 2.2. Cell Culture

Two human osteosarcoma cell lines—HOS (ATCC no. CRL-1543) and 143B (ATCC no. CRL-8303)—were obtained from American Type Culture Collection (Manassas, Va). The cells were cultured and maintained at 37°C in 5% CO_2_ in Eagle's Minimum Essential Medium containing 10% heat-inactivated fetal bovine serum and 5% penicillin/streptomycin (Invitrogen, Carlsbad, Calif). A normal human osteoblast cell line, HOB (C-12720), was obtained and grown in Osteoblast Growth Medium (PromoCell, Heidelberg, Germany). In an attempt to minimize the difference between the human tissues and control osteoblasts, HOB cells were pelleted, fixed in formalin, and embedded in paraffin prior to RNA extraction as previously described (http://web.ncifcrf.gov/rtp/lasp/phl/immunohistochemistry.asp).

### 2.3. RNA Extraction

RNA purification of FFPE samples was performed using RecoverAll Total Nucleic Acid Isolation system (Ambion, Austin, Tex) according to the manufacturer's instructions. In all the sections provided by the pathology department as well as the embedded HOB cells, tumor areas were carefully macrodissected using a scalpel and transferred into a 1.5-mL centrifuge tube. Paraffin was removed by 100% xylene at 50°C for 3 min. The tube was centrifuged, xylene was discarded, and the pellet was washed twice with 100% ethanol followed by air drying. The pellet was digested by protease at 50°C for 15 minutes, and at 80°C for 15 minutes and RNA was purified with filter cartridges. Total RNA of the cell lines (HOS and 143B) was extracted using TRIzol (Invitrogen) according to the manufacturer's instructions. RNA quality and quantity was determined using a NanoDrop ND-1000 spectrophotometer (NanoDrop, Wilmington, Del). Of the 10 samples obtained, 7 had high-quality RNA and were used for further analysis. Four samples were used in the initial miRNA profiling experiments, and 3 were used for validation of the findings.

### 2.4. miRNA Expression Profiling of Cell Lines and Initial Tumor Samples

TaqMan miRNA assays were used to quantify the levels of 762 mature miRNAs in osteosarcoma cell lines, normal human osteoblasts, and 4 tumor samples as described previously [15, 16]. Experimental replicates were performed for all cell lines. Each reverse transcriptase (RT) reaction contained purified total RNA and the TaqMan MicroRNA Megaplex Reverse Transcription Kit (Applied Biosystems, Foster City, Calif) prepared according to the manufacturer's instructions. The reactions were incubated for 30 min at 16°C, 30 min at 42°C, and 5 min at 85°C. The resulting cDNA was then preamplified using Megaplex PreAmp Primers according to manufacturer's instructions. Real-time PCR reactions for each miRNA were performed in a 900-*µ*L reaction mixture that included 9 *µ*L of diluted and preamplified RT product, 450-*µ*L of 2X TaqMan Universal PCR Master Mix, No AmpErase UNG (Applied Biosystems), and 441-*µ*L of nuclease free water. Reactions were incubated in an Applied Biosystems 7900HT Fast Real-Time PCR system in 384-well low-density arrays (TLDAs) at 94.5°C for 10 min, followed by 40 cycles at 97°C for 30 s and 60°C for 1 min.

### 2.5. Confirmation and Validation of Individual miRNAs

After the initial screening, 4 differentially expressed miRNAs were selected, and total RNA was purified from cell lines and the initial 4 tumor samples and was used to perform individual miRNA assays. Each reverse transcriptase (RT) reaction contained total RNA and the TaqMan Individual MicroRNA Reverse Transcription Primer (Applied Biosystems), prepared according to the manufacturer's instructions. The reactions were performed as described in Section 2.4. 

 Real-time PCR reactions for each individual miRNA were performed in a 20-*µ*L reaction mixture that included 1.33-*µ*L of diluted RT product, 1-*µ*L of 20C TaqMan Individual microRNA assay, 10-uL of 2X TaqMan Universal PCR Master Mix, No AmpErase UNG (Applied Biosystems) and 7.67-*µ*L of nuclease free water. Reactions were incubated in triplicate on an Applied Biosystems 7900HT Fast Real-Time PCR system in 96-well plates in the PCR conditions described above. Expression levels of the selected miRNAs were then validated in total RNA from three additional tumor samples that had not previously been tested using the same methodology described here.

### 2.6. Statistical Analysis

Data from miRNA profiling experiments was analyzed using the RealTime StatMiner software (Version 4.0, Integromics). The negative Ct values of all miRNA probes were median centered by sample and then normalized against the endogenous control Mammalian U6. The expression values were subjected to hierarchical clustering by Pearson's correlation by using Multiexperiment Viewer (Version 4.5.1, http://www.tm4.org/mev/). For individual assay confirmation and validations, Student's *t*-tests were used to assess the statistical significance between the relative miRNA expression between tumors and control. For all analyses, a two-tailed value of *P* < 0.05 was considered significant.

## 3. Results

### 3.1. Differential Expression of miRNAs in Osteosarcoma

**Figure 1:**
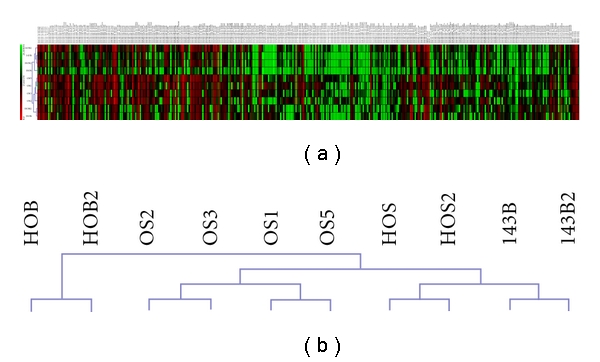
Heat Map showing unsupervised hierarchical clustering performed using Pearson's correlation (Multiexperiment Viewer, Version 4.5.1). Relative expression values from normal osteoblasts (HOB), 2 osteosarcoma cell lines (HOS and 143B), and 4 human tumors samples (OS 1, 2, 3, and 5) are represented in the main tree (a). A higher magnification of the column dendrogram showing that each group clusters together suggesting that the samples have similar miRNA expression profiles (b).

Real-time qPCR was used to evaluate the relative expression of 762 mature miRNA expression levels in 4 human tumors and 2 osteosarcoma cell lines (HOS and 143B) compared to a normal human osteoblast cell line (HOB). Unsupervised hierarchical clustering was performed using the relative expression values for all samples and all miRNA probes. Based on their expression values, each group (normal osteoblasts, human tumors, and osteosarcoma cell lines) clustered together, thus confirming similar miRNA expression profiles (Figure 1). Additionally, the tumor samples and osteosarcoma cell lines clustered independently from the normal human osteoblasts.

### 3.2. Pairwise Comparison of Test Groups

RealTime StatMiner software was used to compare the expression profiles between human tumors and normal human osteoblasts. This analysis revealed 22 significantly differentially expressed miRNAs (*P* < 0.05) (Table 1). Two additional comparisons were done. Osteosarcoma cell lines were compared to normal osteoblasts, and 5 individual miRNAs that matched those in the original list were identified. Finally, osteosarcoma cell lines and human tumors were compared, and 14 out of the 22 previously identified miRNAs exhibited statistically significant differential expression.

### 3.3. Selection of Candidate miRNAs for Validation

Of the 22 miRNAs initially identified, 4 were selected for individual confirmation (miR-135b, miR-150, miR-542-5p and miR-652) based upon the fact that they were differentially expressed in both comparisons of human tumor and osteosarcoma cell lines when compared to osteoblasts. Furthermore, 3 of these candidates (miR-135b, miR-542-5p, and miR-652) had no expression in tumor samples and osteosarcoma cell lines, which minimizes the likelihood that expression differences are a result of differences between immortalized cell lines and heterogeneous tumor samples. Despite the verified difference in expression of miR-150 comparing tumors and cell lines, this candidate miRNA was selected for validation given the amplitude of expression differences observed when tumors were compared to osteoblasts.

### 3.4. Confirmation of Candidate miRNAs in the Original Samples

Individual miRNA assays were performed on total RNA from the initial tumor samples to determine the expression levels of miR-135b, miR-150, miR-452-5p and miR-652 when compared to normal human osteoblasts. All 4 miRNAs were overexpressed in tumors when compared to normal osteoblasts as shown in Table 2.

### 3.5. Validation of Candidate miRNAs in the Test Samples

**Figure 2:**
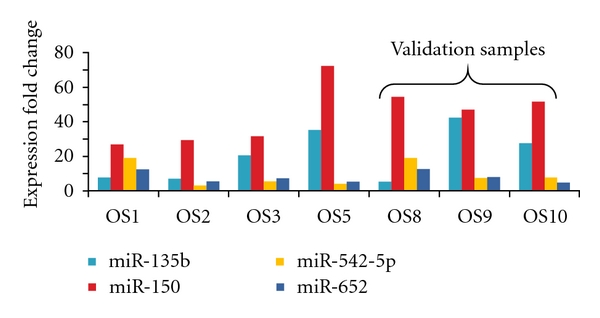
Overexpression of selected miRNAs in osteosarcoma tumor samples. Individual miRNA assays were performed with the 4 initial samples confirming the differential expression of 4 miRNAs that were identified when compared to normal osteoblasts in the first screening. A “test set” comprising of three additional tumors was used to further validate these findings.

Total RNA from three additional tumor samples that had not previously been analyzed was used for individual miRNA assays of the 4 candidate miRNAs. For all 4 probes, the miRNAs were overexpressed when compared to normal human osteoblasts in a similar pattern to that of the first 4 tumors samples. The expression levels for all 7 samples are shown in Figure 2.

## 4. Discussion

With the objective of identifying miRNAs that are important in osteosarcoma tumorigenesis, we have performed miRNA expression profiling of two osteosarcoma cells lines as well as 4 FFPE human tumor samples using TLDA arrays. We profiled 762 miRNAs and identified 22 that are differentially expressed in osteosarcoma (Table 1). Furthermore, we have confirmed and validated 4 candidate miRNAs in the initial cohort of tumor samples, and in a test set of 3 additional tumor samples. To our knowledge, our study is among the first to perform TaqMan array based miRNA profiling in osteosarcoma samples using FFPE samples.

In our study, we were able to reliably extract miRNAs from FFPE tissues of human osteosarcoma. Several groups have already discussed the utility and reliability of using FFPE samples for microRNA expression analyses [17, 18]. One of the barriers for basic research in osteosarcoma is the fact that most tumor tissues are decalcified prior to histological analyses; as a result, the nucleic acids are degraded. However, we were able to successfully perform our analyses on nondecalcified tumor blocks obtained from primary biopsies.

In the tumor samples used in our study, miR-135b was one of the miRs found to be significantly overexpressed in human osteosarcoma when compared to normal osteoblasts. Recently, expression profiling studies in both colon cancer and prostate cancer have shown consistent overexpression of miR-135b in affected tissues [19, 20]. Additionally, a recent study has demonstrated that low levels of miR-135b are important for the normal development and mineralization of osteoblasts. Overexpression of miR-135b resulted in abnormal mineralization, and differentiation of unrestricted somatic stem cells [21]. We hypothesize that high levels of miR-135b may inhibit normal differentiation of stem cells into osteoblasts and this might explain the abnormal growth of osteosarcoma cells. However, further analyses exploring the targets of miR-135b are warranted. 

Other groups have already shown that the expression of miR-150, a hematopoietic specific miRNA, is important in the differentiation of B and T lymphocytes in normal hematopoiesis [22]. The aberrant expression of miR-150 in solid tumors has been recently explored by Wu et al., and they reported high levels of miR-150 in gastric cancer tissues. This analysis has confirmed that overexpression of miR-150 is able to promote gastric cancer proliferation both *in vitro* and *in vivo * [23]. In our study, we have identified a nearly 50-fold overexpression of miR-150 in osteosarcoma samples when compared to normal osteoblasts. Importantly, the proapoptotic EGR2 (early growth response 2) and the P2X7 receptor have been confirmed as the targets of miR-150 [23, 24]. The P2X7 receptor is involved in signal transduction and has been well characterized as a promoter of apoptosis in both osteoblasts and osteoclasts [25]. Based on these analyses and the results presented here, we hypothesize that upregulation of miR-150 may downregulate the P2X7 receptor resulting in uncontrolled growth of osteosarcoma cells. 

The two other microRNAs that were overexpressed and confirmed in this study, miR-542-5p and miR-652, have only a few reports in the literature. Recently, a group has reported the association of miR-542-5p overexpression in favorable histology, MYCN nonamplified neuroblastoma samples [26, 27]. No data are available in the literature regarding miR-652 and cancer thus far; however, bioinformatic predictions suggest that this miRNA has more than 800 potential gene targets. The findings of our study with regard to miR-542-4p and miR-652 will require further investigation in osteosarcoma.

Results of our miRNA profiling experiments revealed 22 differentially expressed miRNAs. Given the scope of this study, validation was only done for 4 individual miRNAs. However, many of the other identified miRNAs may be important in osteosarcoma tumorigenesis. One such example is miR-210, which was found to be significantly elevated in our tumor samples and it has been well studied in other cancers. miR-210 is a key player in the hypoxic response, and it has been found to be upregulated in all cell types tested thus far in hypoxic conditions [28]. Recently, miR-210 was found to be a positive regulator of osteoblastic differentiation through inhibition of *AcvR1b* (activin A receptor type 1B) [29]. Given the findings of this study, the role of miR-210 in osteosarcoma should be further investigated. 

The results of our study share some similarities with a paper recently published by Maire et al. in which miRNA expression patterns in frozen osteosarcoma tumor specimens were compared with osteoblasts. Further, gene expression data on the same specimens was also used to explore mechanisms of miRNA deregulation in osteosarcoma [30]. Amongst others, the authors report overexpression of miR-126, miR-142-3p and miR-451 as was also shown in our data (Table 1). In some cases, miRNA-overexpression corresponded to gains in the miRNA coding sequences in areas of known chromosomal alterations in osteosarcoma. Additionally, several underexpressed miRNAs were also reported [30]. Taken together with our data, this suggests a critical role for miRNA expression with and without DNA copy alterations in osteosarcoma.

Our study has some limitations that warrant discussion. The number of patient samples is low with only 7 of the 10 samples containing RNA suitable for analysis. The remaining 3 samples had a significant amount of necrosis on the representative H&E section, which likely explained the poor RNA quality. Confirmation of these findings in a larger cohort of tumor samples would be of importance. We also acknowledge that the normal control selected for this study is a normal adult human osteoblasts cell line. Though some of the miRNA differences may be inherent differences between cell lines and human tissue, we attempted to minimize this confounder by simultaneously using tumor cells lines as well as tissue samples to choose candidate miRNAs for confirmation. For future studies, pediatric osteoblasts and/or sections of normal bone enriched for osteoblasts should be considered as normal controls. 

In conclusion, our study is one of the first to use osteosarcoma samples in FFPE to perform miRNA expression analysis by TaqMan Real-Time PCR in large scale and to compare them to normal human osteoblasts. Using this strategy, we were able to identify twenty-two differentially expressed microRNAs, and 4 (miR-135b, miR-150, miR-542-5p and miR-652) were selected for individual confirmations. Overexpression of the selected candidate miRNAs was further validated in 3 additional tumor FFPE samples. In our samples, miR-135b was significantly overexpressed, and it has already been shown to have a role in normal osteoblastic differentiation; high levels of miR-135b result in abnormal mineralization. Additionally, miR-150 may contribute to osteosarcoma tumorigenesis by inhibition of proapoptotic genes as shown in other cancers. Finally, several other differentially expressed microRNAs were uncovered in our study (e.g., miR-210) and warrant further evaluation in osteosarcoma tumorigenesis.

## Figures and Tables

**Table 1:** Fold change differences from 22 miRNAs comparing human tumor samples, osteosarcoma cell lines, and normal osteoblasts.

miRNA	Tumors versus control*	Cell lines versus control*	Tumors versus cell lines*
hsa-miR-126	652.38		65.02
hsa-miR-135b	286.45	137.51	
hsa-miR-140-3p	116.06		43.17
hsa-miR-140-5p	19.91		87.88
hsa-miR-142-3p	3103.47		3589.12
hsa-miR-148a	23.30		115.05
hsa-miR-150	15660.58	88.72	176.50
hsa-miR-18a	88.84		
hsa-miR-194	583.46		63.78
hsa-miR-198	−33.11	−714.28	21.00
hsa-miR-200b	230.73		
hsa-miR-210	2340.79		52.98
hsa-miR-223	215.02		27387.46
hsa-miR-301b	71.53		
hsa-miR-450a	389.49		18.64
hsa-miR-451	601.47		
hsa-miR-454	97.52		
hsa-miR-455	1433.78		68.88
hsa-miR-511	185.41		384.98
hsa-miR-542-5p	497.64	62.86	
hsa-miR-652	348.87	458.92	
hsa-miR-660	67.65		39.31

*P ≤ 0.05 for all comparisons.

**Table 2:** Mean expression fold changes of candidate miRNAs in 7 human tumors as compared to normal osteoblasts.

miRNA	Average fold change	Standard deviation
hsa-miR-135b*	≥20.88	14.81
hsa-miR-150	44.77	16.50
hsa-miR-542-5p	9.47	6.77
hsa-miR-652	8.00	3.33

*No miR-135b was detected in the control osteoblasts.
